# Effect of Multiple Mechanical Recycling Cycles on the Structure and Properties of PHBV Biocomposites Filled with Spent Coffee Grounds (SCG)

**DOI:** 10.3390/ma18235368

**Published:** 2025-11-28

**Authors:** Grzegorz Janowski, Wiesław Frącz, Łukasz Bąk, Janusz W. Sikora, Adam Tomczyk, Grażyna Mrówka-Nowotnik, Beata Mossety-Leszczak, Beata Pawłowska

**Affiliations:** 1 Department of Materials Forming and Processing, Rzeszow University of Technology, Powstancow Warszawy 8, 35-959 Rzeszow, Poland; 2 Department of Technology and Polymer Processing, Lublin University of Technology, Nadbystrzycka 36, 20-618 Lublin, Poland; 3 Department of Mechanics and Applied Computer Science, Faculty of Mechanical Engineering, Białystok University of Technology, Wiejska 45C Str., 15-351 Białystok, Poland; 4 Department of Material Science, Rzeszow University of Technology, Al. Powstańców Warszawy 12, 35-959 Rzeszow, Poland; 5 Department of Industrial and Materials Chemistry, Rzeszow University of Technology, Al. Powstańców Warszawy 6, 35-959 Rzeszów, Poland

**Keywords:** PHBV, spent coffee grounds (SCG), biocomposites, mechanical recycling, mechanical properties

## Abstract

The growing demand for sustainable materials in a circular economy necessitates the evaluation of the recyclability of biodegradable composites. This study aims to investigate the effect of multiple mechanical recycling cycles on the properties of a poly(3-hydroxybutyrate-co-3-hydroxyvalerate) (PHBV) biocomposite containing 45 wt% spent coffee grounds (SCG). The material was produced via extrusion and injection molding, followed by five consecutive recycling cycles under controlled processing parameters. Changes in mechanical properties (tensile strength, elastic modulus, elongation at break, hardness, and impact tensile strength), processing shrinkage, thermal structure (DSC), and microstructure (SEM) were evaluated. The results revealed a gradual increase in PHBV crystallinity, confirmed by DSC analysis. Consequently, the changes in mechanical properties were significant; specifically, the elastic modulus increased by approximately 9.6% and hardness improved, whereas elongation at break decreased by approx. 18% and impact strength declined, indicating a transition towards a stiffer but more brittle material. SEM observations suggested microstructural evolution with reduced agglomerates after subsequent cycles and the predominance of a brittle fracture mechanism. Linear shrinkage in the flow direction remained stable, whereas changes in thickness shrinkage correlated with the formation of micropores. The findings demonstrate that PHBV-SCG biocomposites maintain adequate mechanical and processing performance even after five recycling cycles, highlighting their potential for applications within a circular economy framework.

## 1. Introduction

Poly(3-hydroxybutyrate-co-3-hydroxyvalerate) (PHBV) is a biodegradable biopolymer from the polyhydroxyalkanoate (PHA) family, produced from renewable raw materials through microbial fermentation. It is fully biodegradable; however, its practical applications are limited by brittleness, low thermal stability, and a narrow processing window, as its melting temperature is close to its thermal degradation temperature [1]. To reduce costs and improve processing performance, PHBV is often reinforced with lignocellulosic fillers of plant origin. Such solutions maintain the material’s biodegradability, enhance stiffness, and lower the proportion of the costly polymer in the composite. In this context, spent coffee grounds (SCG)—a widely available bio-waste rich in lignin, lipids, and carbohydrates—are a particularly attractive filler [2,3,4,5].

Studies have shown that the addition of SCG increases the stiffness of PHBV composites, although it does not improve their tensile strength due to weak adhesion between the organic filler and the polymer matrix [1,6,7]. Notably, the presence of SCG enables processing of the biocomposites at lower temperatures (~170 °C), which is advantageous for thermally sensitive PHBV [6,7]. In comparison, other lignocellulosic fillers, such as wood flour or hemp fibers, require higher processing temperatures (around 180–190 °C) [1]. The PHBV/SCG material itself can be considered sustainable, as it combines a biodegradable polymer matrix with a waste-derived organic filler. However, its end-of-life management and potential for efficient recycling remain important considerations.

Three primary strategies are generally distinguished for managing bioplastic and SCG waste:

- Mechanical recycling—involves reprocessing the polymer without altering its chemical structure, encompassing steps such as sorting, cleaning, shredding, remelting, and forming into new products. Recent advancements highlight techniques such as Material Extrusion (MEX) based additive manufacturing, which allows for the fabrication of customized parts from waste filaments but may be prone to porosity and internal defects [8]. In contrast, injection molding enables high-volume production and typically yields superior mechanical performance, such as increased tensile strength and stiffness, by minimizing voids through high-pressure processing compared to MEX-printed parts [9]. PHBV-based composites with natural fillers have been shown to maintain good properties even after multiple processing cycles [10].
- Chemical recycling—enables the depolymerization of PHBV into monomers or other chemical compounds, though this method remains costly and challenging to implement on an industrial scale [5,11,12,13].
- Biological recycling—uses biodegradation processes such as aerobic composting or anaerobic fermentation. Both PHBV and SCG are fully biodegradable and can be effectively managed through these approaches [6,11,12,13].

The choice of recycling strategy depends on the material’s properties, available infrastructure, and the economic feasibility of the selected method.

The mechanical recycling of PHBV does not differ significantly from processes applied to conventional plastics. With properly optimized processing conditions (temperature, time, pressure, humidity), this biopolymer can be reused multiple times without a significant loss of mechanical or functional properties [10]. Moreover, the presence of natural fillers such as SCG can further facilitate processing. Lignin compounds present in SCG reduce processing shrinkage, improve dimensional stability of molded parts, and minimize the risk of deformation during cooling [1,7].

The addition of SCG also has a beneficial effect on the thermal parameters of processing—it enables a reduction in the composite’s softening temperature, minimizing the risk of PHBV degradation during remelting [6,7]. As a result, PHBV/SCG biocomposites can be processed at lower temperatures than comparable composites containing other lignocellulosic fillers (e.g., hemp fibers or wood flour), which enhances their resistance to multiple processing cycles and reduces energy consumption during manufacturing [2,7]. Studies have also shown that repeated processing of PHBV with natural fillers promotes improved system homogenization—filler particles become increasingly well dispersed, and interfacial contact with the polymer matrix is strengthened [6,10]. In the case of SCG-based composites, it has been observed that the progressively fragmented particles integrate more readily with the PHBV matrix during subsequent processing cycles, improving the material’s mechanical performance and reducing internal stress concentrations [1,6,14]. Additionally, the use of chemically or biologically modified coffee grounds (e.g., through alkalization, silanization, or enzymatic treatment) increases interfacial compatibility, resulting in improved adhesion and enhanced composite durability [15,16].

The main limitation of PHBV mechanical recycling is its relatively low thermal stability. PHBV begins to degrade at temperatures above ~170–180 °C, meaning that even minor temperature overshoots can lead to chain depolymerization and the emission of degradation products (e.g., crotonic acid) [2,17]. This results in reduced molecular weight and deterioration of mechanical properties, particularly after several processing cycles. In addition, degradation can also occur under aerobic conditions, leading to oxidation of chain ends and increasing the material’s susceptibility to further biological breakdown [2,10]. SCG, despite its many advantages, also undergoes degradation. During multiple processing cycles, SCG particles can undergo charring due to overheating, which reduces their reinforcing effect [2,7,12]. This phenomenon may become more pronounced in subsequent recycling cycles, especially if processing parameters are not carefully optimized. Excessive SCG content is another constraint—at concentrations above ~30%, the filler tends to agglomerate, weakening the composite structure and reducing its mechanical performance [14,15,16]. Another significant issue is the hygroscopicity of both PHBV and SCG. These materials readily absorb moisture from the environment, which during processing can lead to polyester hydrolysis. The presence of moisture causes water vapor formation, voids, and even porosity in the final product. Therefore, material intended for reprocessing must be thoroughly dried (preferably under vacuum or in a dry-gas atmosphere) prior to remelting [1,4]. A further limitation is the gradual loss of interfacial compatibility. If the original composite contained additives (e.g., crosslinkers, compatibilizers, stabilizers), their effectiveness may decrease with each processing cycle due to thermal degradation or leaching from the matrix [12,18,19]. To counteract property deterioration, rejuvenation strategies are recommended, such as blending with virgin polymer, incorporating stabilizers (e.g., antioxidants, UV stabilizers), or using chain extenders to restore polymer chain length [12,18,19].

The mechanical recycling of PHBV/SCG biocomposites holds significant potential from a sustainable development perspective. Combining a biodegradable polymer matrix with a waste-derived lignocellulosic filler not only enables the recovery of valuable resources but also substantially reduces processing costs and the product’s carbon footprint [2,5]. Further optimization of processing parameters, incorporation of additives to enhance material durability (e.g., stabilizers, antioxidants, chain extenders), and the development of post-recycling material conditioning technologies (e.g., reactive crosslinking agents, biological modification of SCG, or surface activation treatments) could significantly improve the quality and longevity of recycled products, thereby increasing the number of achievable closed-loop cycles [6,14,15,16,17,18]. The physicochemical properties of poly(3-hydroxybutyrate-co-3-hydroxyvalerate) reinforced with spent coffee grounds play a decisive role in selecting recycling strategies and designing effective recovery processes. Key considerations include biodegradability, thermal stability, and the mechanical performance of the composite.

Both PHBV and SCG are biodegradable materials, which enables their disposal through biological recycling processes such as composting. However, it is important to note that full PHBV biodegradation occurs only under conditions favorable to specific microorganisms, typically at elevated temperatures, as in industrial composting facilities [8,14]. The presence of natural fillers such as SCG accelerates degradation slightly compared to the polymer matrix alone, increasing material porosity and facilitating microbial access to the PHBV structure [7]. Organic compounds present in coffee grounds (e.g., residual sugars and proteins) may promote the initiation of biodegradation, while the lignin content slows down decomposition—lignin residues may persist in compost for an extended period before undergoing full humification [2,6,7]. High biodegradability is advantageous in preventing the accumulation of persistent waste; however, it also necessitates well-organized collection and processing systems to avoid uncontrolled degradation during storage or transportation. PHBV exhibits relatively good stability under dry, room-temperature conditions, allowing products to be safely used for several months [14]. Nevertheless, each mechanical recycling cycle can introduce defects such as oxidized chain ends, which facilitate microbial attack and shorten the degradation time at the end of the product’s life [10,19]. This phenomenon is an important consideration when designing the service life of PHBV/SCG-based products.

As reported in the literature, PHBV exhibits limited thermal stability, undergoing thermal chain scission above approximately 180 °C, accompanied by the release of degradation products such as crotonic acid [2]. The addition of SCG in moderate amounts can influence the thermal properties of the composite. On one hand, the filler itself has limited thermal resistance—its cellulose–hemicellulose structure begins to char and degrade at temperatures above ~200 °C [2,6,13]. On the other hand, the lignin present in SCG can act as a stabilizing agent for PHBV by restricting chain mobility and slowing depolymerization processes [7,13]. Differential Scanning Calorimetry (DSC) and Thermogravimetric Analysis (TGA) results indicate that the incorporation of 15% SCG does not significantly reduce the thermal decomposition temperature of PHBV. In some cases, even higher processing temperatures were required to ensure proper matrix plasticization and uniform filler dispersion [2,13]. At higher SCG contents, the polymer softens earlier, allowing processing at lower temperatures, which minimizes the risk of thermal degradation of both the polymer and the filler [2,6,7]. In the context of mechanical recycling, the relatively low thermal resistance of PHBV necessitates precise control of processing parameters, particularly heating temperature. Conversely, for chemical recycling, PHBV’s susceptibility to depolymerization may be advantageous, as its breakdown can occur under milder conditions compared to more durable polyesters such as PET [13,17].

A key limitation of PHBV as a structural material is its brittleness—despite its high stiffness, it exhibits low ductility. The addition of SCG further increases the composite’s elastic modulus while reducing its elongation at break [2,7,19]. Even after the first thermomechanical processing cycle, microcracks may appear at the polymer–filler interfaces. However, studies on analogous PHBV composites, such as those reinforced with buckwheat hulls, have shown that repeated processing cycles can lead to improved filler particle dispersion, structural homogenization, and even increased hardness, despite a reduction in impact strength [10,20]. In the case of coffee grounds, their initial tendency to form agglomerates can result in localized stress concentrations. Successive extrusion and molding cycles promote agglomerate breakdown and improved phase distribution, which helps stabilize the mechanical properties of the material [2,7,21]. When high strength of the final product is required, the use of performance-enhancing additives (e.g., chain extenders or compatibilizers) may be beneficial to improve adhesion between SCG and the polymer matrix [6,19,21].

Another important aspect is processing shrinkage, which affects the dimensional stability of products. PHBV exhibits relatively high shrinkage (~1.2%), whereas the addition of SCG significantly reduces it, which is advantageous for both primary products and those made from recycled material [6,14]. Lower shrinkage translates to reduced internal stresses and a lower risk of product deformation and cracking. Literature also indicates that properly selected additives (e.g., peroxides, stabilizers, or crosslinking compatibilizers) can effectively enhance the mechanical properties of PHBV/SCG recyclates, even at high filler loadings [17,19,21].

Previous literature studies highlight the potential of SCG as a filler in biocomposites based on various polymers, including PHBV. However, most research focuses on lower filler contents (typically 10–20%) and single-cycle properties. There is a notable lack of reports on the feasibility of mechanical recycling of PHBV-based biocomposites with high waste filler loadings. Therefore, this study aims to address this research gap by analyzing a PHBV-based biocomposite containing 45 wt% SCG. This specific loading level was selected to maximize the valorization of waste material and significantly reduce the consumption of the primary polymer matrix, addressing both economic and environmental goals. While challenging compared to lower filler contents typical in the literature, this approach allows for a robust assessment of the biocomposite’s recycling potential in high-sustainability applications, providing insights into material stability at the upper limits of filler incorporation. To achieve this, the material was subjected to up to five successive reprocessing cycles using injection molding. Both mechanical, functional, and processing properties, as well as thermal stability and microstructural changes, were evaluated in the context of repeated recycling. This approach provides a comprehensive assessment of the PHBV/SCG biocomposite’s resistance to reprocessing and determines its application potential in a circular economy.

## 2. Materials and Methods

### 2.1. Materials

The polymer matrix used in this study was PHBV, supplied under the trade name ENMAT Y1000 by Tianan Biologic Materials Co., Ltd. (Ningbo, China) [22]. The material was delivered in powder form, which facilitated accurate dosing and uniform mixing with the SCG filler. According to the manufacturer’s technical data sheet, ENMAT Y1000 is characterized by a density of 1250 kg·m^−3^, a melt flow index (MFI) of 1–5 g/10 min measured at 190 °C under a 2.16 kg load (ISO 1133:1997 [23]), a Vicat softening temperature of 166 °C (ISO 306:1994 [24]), and a heat deflection temperature (HDT) of 157–165 °C (ISO 75-2:2003 [25]). These properties are typical for PHBV and enable its processing using conventional thermoplastic technologies, including extrusion and injection molding, which is essential for evaluating the impact of repeated mechanical recycling in the present study.

SCG was used as the filler, representing a lignocellulosic waste fraction generated after the coffee brewing process. To minimize variability in raw material properties, SCG was sourced exclusively from a single coffee brand—Jacobs Kronung (Jacobs Douwe Egberts, Amsterdam, Netherlands). The material was collected from restaurants and cafés, then dried for 24 h at 60 °C to reduce moisture content and stabilize its material characteristics. After drying, the SCG was stored in dry conditions and protected from moisture until processing.

The filler content in the biocomposite was set at 45 wt%. This high loading was chosen to evaluate the material’s performance at the upper limits of filler incorporation subjected to multiple recycling cycles. In the subsequent sections of this paper, the following notation system was adopted for the PHBV-SCG (45 wt%) biocomposite samples, as summarized in Table 1.

### 2.2. Production of Biocomposite by Extrusion Process

Before processing, both the polymer matrix (PHBV) and the SCG were dried at 90 °C for 3 h to remove moisture and stabilize the extrusion conditions.

The PHBV–SCG biocomposite was produced by extrusion followed by cold pelletizing. The experiments were carried out using a research setup consisting of a ZAMAK EHP-25E single-screw extruder (Zamak Mercator, Skawina, Poland), a cooling section, and a pelletizer. The extruder was equipped with a barrel of 25 mm diameter and an L/D ratio of 27, featuring three independent heating zones and a die head with a 2 mm nozzle.

The pre-dried feed material was gravity-fed into the hopper zone, from where it was conveyed and plasticized in the barrel and screw system. During processing, simultaneous plasticization of the PHBV matrix and homogenization with the SCG filler occurred, ensuring a uniform distribution of particles throughout the material. The screw rotation speed was kept constant at 100 rpm, which was chosen based on preliminary trials to ensure adequate mixing of the high filler load without generating excessive shear heat. The detailed temperature settings for each zone and the die head are summarized in Table 2. These temperatures were established based on the polymer manufacturer’s data but were experimentally optimized to the lowest possible range to prevent thermal degradation of the sensitive PHBV matrix and the lignocellulosic filler.

The extrusion process was assessed as stable. The melt pressure was measured at the screw tip, while the screw load was continuously monitored, enabling real-time control of processing conditions. Both parameters showed consistent values without sudden fluctuations that would indicate flow disturbances or blockages in the plastification system. The temperatures in each zone were set according to the controller settings, and their stability ensured reproducible thermal conditions throughout the process. The extruded strands exhibited a uniform color and smooth surface, confirming the absence of localized overheating and polymer degradation.

Unlike conventional processes, the extrudate was not immersed in a water cooling bath but was dried using a stream of cold air. This approach minimized moisture absorption by the composite, which could otherwise degrade its properties during subsequent processing steps (particularly injection molding). As a result, a dry and stable feed material was obtained, ensuring improved suitability for subsequent recycling cycles.

The cooled strands were then fed into a G 13/32 pelletizer (Zamak Mercator, Skawina, Poland), where they were cut into pellets measuring 2–5 mm in length. The pellets exhibited a uniform shape and minimal dimensional variation, which facilitated subsequent dosing. Incorporating the pelletizing stage was technologically significant, as it enabled the production of a homogeneous feedstock with consistent geometry, typical of industrial injection molding processes. This approach not only supported laboratory-scale testing but also reproduced real industrial processing conditions, thereby enhancing the reliability of the obtained results.

**Figure 1:**
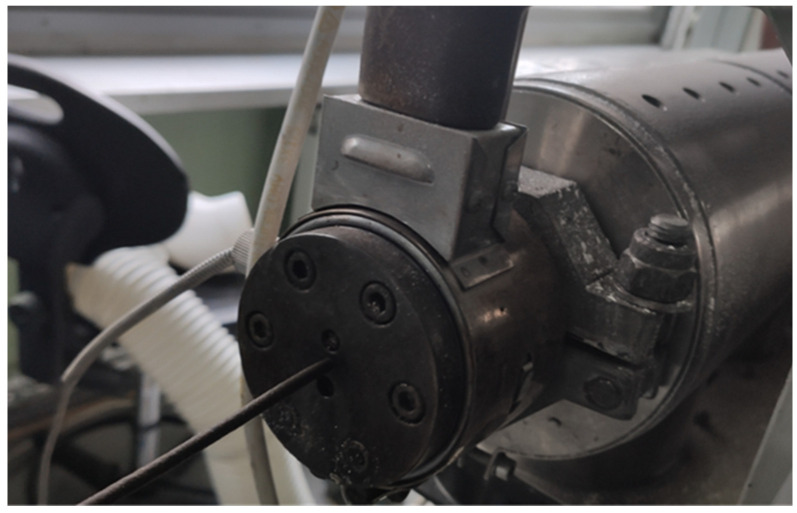
Extrusion of the PHBV–SCG composite.

**Figure 2:**
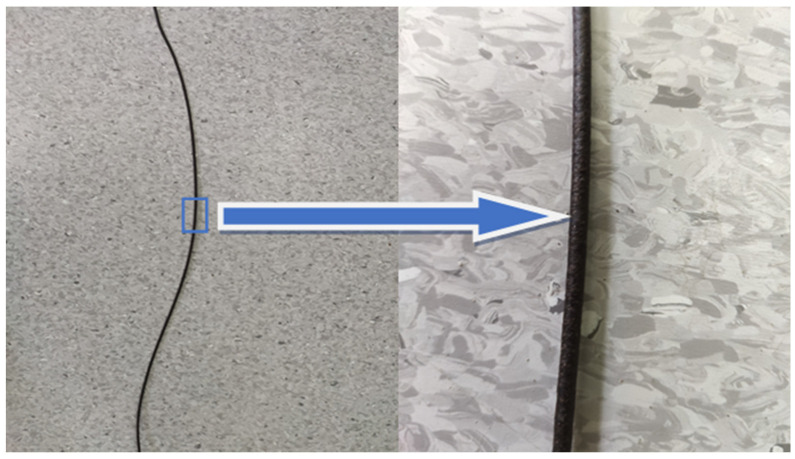
Presentation of the biocomposite extrudate. The blue rectangle marks the section selected for magnification, and the arrow points to the location of the magnified image.

**Figure 3:**
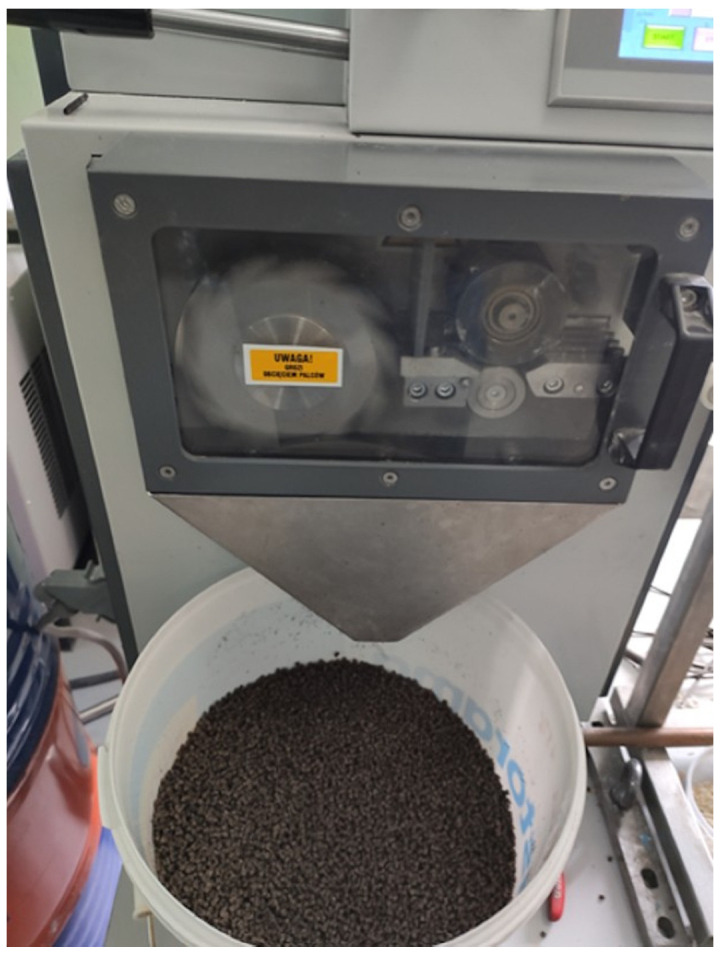
Pelletizer with biocomposite pellets.

Figure 1 shows the extrudate exiting the extrusion die, Figure 2 presents a section of the extrudate after shaping in the cooling section, and Figure 3 illustrates the pelletizing process of the biocomposite strands.

### 2.3. Injection Molding and Recycling

A DrBoy 55E hydraulic injection molding machine (Dr. Boy GmbH and Co. KG, Neustadt, Germany) equipped with a Priamus system (Priamus System Technologies, Schaffhausen, Switzerland) was used to produce specimens for mechanical testing. The Priamus system enabled real-time monitoring of processing parameters and active control of the injection molding process. This setup allowed precise regulation of variables such as cavity pressure and temperature, which is particularly important for biocomposites with a high filler content due to the risk of structural inhomogeneity and the sensitivity of the polymer matrix to overheating.

The injection molding machine was equipped with a standard plastification unit featuring a 32 mm screw, enabling injections with volumes ranging from 2 to 96 cm^3^ and a maximum clamping force of 550 kN. A mold with interchangeable inserts was used for the tests, featuring cavities designed to produce paddle-shaped specimens in accordance with the EN ISO 527-1:2012 [26] standard for uniaxial tensile testing. This setup ensured specimens with consistent geometry, enabling direct comparison of the composite’s mechanical properties after successive processing cycles.

Injection molding process parameters, such as cylinder zone temperatures, injection pressure, holding time, and mold temperature, were optimized for processing PHBV–SCG biocomposites and are summarized in Table 3. The obtained biocomposite specimens were tested, after which the material underwent five successive reprocessing cycles using the same injection molding technology. Specimens produced in each subsequent reprocessing cycle were also subjected to the tests described in the following sections of this study.

Before each injection molding cycle, the material was dried in a laboratory vacuum dryer to minimize moisture content in the biocomposite granulate. This step was essential due to the high hygroscopicity of PHBV and the SCG fraction, which can absorb moisture under elevated humidity conditions, leading to agglomeration and structural inconsistencies in the material. Excess water in the granulate also increases the risk of processing defects, including air bubbles within the cross-section of molded parts, as well as reduced interfacial adhesion. Drying was therefore crucial for achieving a homogeneous composite structure and improving the reproducibility of specimen manufacturing parameters.

The drying process was carried out in a Chemland DZ-2BC laboratory vacuum dryer (Stargard, Poland) with a maximum heating power of 1400 W and a chamber capacity of 52 L, paired with a Value V-i120SV vacuum pump with a flow rate of 51 L/min. The samples were dried at 90 °C for 6 h under a chamber vacuum of 0.02 MPa. These conditions effectively removed moisture from both the polymer phase and the lignocellulosic filler.

**Figure 4:**
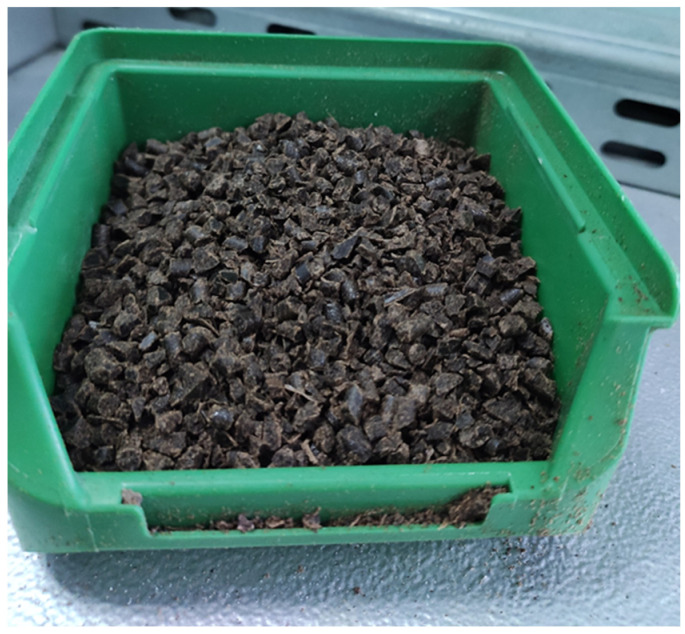
Regranulate/shredded composite intended for reprocessing.

Preparation of the biocomposite granulate before each cycle also included crushing the previously molded parts for regranulation. This operation was performed using a Wanner C17.26 SV plastic granulator (Wertheim-Reicholzheim, Germany). The device featured a working chamber measuring 170 × 260 mm^2^, a feed capacity of 6 L, and was equipped with 12 rotary blades and two fixed blades, along with a screen with 5 mm diameter holes. This setup enabled the production of granulate with uniform particle size and a suitable fraction for reprocessing (Figure 4).

The reprocessing of molded parts via injection molding required adjustment of the machine’s set parameters for each subsequent recycling cycle. This necessity arose from the fact that PHBV–SCG biocomposites, after repeated melt processing, exhibit changed melt rheology and, consequently, different flow behavior within the mold cavity. To obtain molded parts with consistent geometric and dimensional quality, it was necessary to adjust cylinder zone temperatures, injection pressure and speed, as well as holding time. The detailed technological parameters applied during injection molding in successive reprocessing cycles are summarized in Table 4.

In addition, proper optimization of processing parameters minimized the risk of manufacturing defects such as sink marks, shrinkage voids, gas bubbles, or incomplete mold filling. In practice, adjusting the processing conditions made it possible to reproduce molded parts with quality comparable to those produced in the first cycle (0×), despite the progressive changes in the material’s physicochemical properties resulting from its processing history.

### 2.4. Methods

The experimental design was structured to monitor the progressive changes in functional properties (mechanical performance, shrinkage) across all recycling cycles. Detailed structural (SEM) and thermal analyses (DSC) were specifically conducted for the initial (0x) and final (5x) cycles to identify the cumulative effects of degradation and microstructural evolution responsible for the observed trends. The summary of processing stages and the specific tests performed on each sample series is presented in Table 5.

To evaluate the microstructure of the specimen fracture surfaces, a HITACHI S-3400N scanning electron microscope (SEM) (Hitachi, Tokyo, Japan) was used. Observations were conducted at magnifications of 125×, 500×, and 3000×, enabling analysis of the adhesion quality between the PHBV polymer matrix and the SCG filler, as well as the identification of processing defects such as voids or agglomerates. For each material type, three specimens were analyzed, and representative images are presented in this study.

Thermal property testing was performed using a DSC differential scanning calorimeter (Mettler-Toledo, Greifensee, Switzerland) cooled with liquid nitrogen. The instrument was calibrated with zinc and indium standards, allowing precise determination of phase transition temperatures. Analyses were conducted in hermetically sealed 40 μL aluminum pans under a nitrogen atmosphere at a constant flow rate of 60 mL·min^−1^, following the EN ISO 11357-1:2016 [27] standard. The temperature program ranged from −50 °C to 250 °C with a constant heating rate of 10 K·min^−1^.

Mechanical strength of the tested biocomposites was evaluated using a Zwick Z030 universal testing machine (Zwick/Roell, Ulm, Germany). Uniaxial tensile tests were performed according to the EN ISO 527-1:2012 standard on dumbbell-shaped specimens. The obtained stress–strain curves were used to determine key mechanical parameters, including tensile strength, Young’s modulus, and elongation at break.

The hardness of the biocomposites was determined using the ball indentation method in accordance with the EN ISO 2039-1:2003 [28] standard. Measurements were taken in two characteristic areas of the tensile specimens: the gauge section (area A) and the grip section (area B). A Zwick 3106 hardness tester (Zwick/Roell, Ulm, Germany) was used, enabling assessment of local variations in mechanical properties across different specimen regions.

Fracture resistance under dynamic loading was evaluated using an impact tensile test according to the EN ISO 8256:2004 [29] standard. A CEAST 9050 pendulum impact tester (Instron, Norwood, MA, USA) was employed to accurately determine the energy absorbed by the specimen during sudden loading.

The linear shrinkage of dumbbell-shaped specimens was determined in accordance with the EN ISO 294-4:2001 [30] standard, enabling evaluation of dimensional changes resulting from processing and cooling.

All mechanical property tests and shrinkage measurements were conducted on 10 specimens for each material type, ensuring reliable experimental results. Mean values and standard deviations were calculated from the collected data and presented in the form of graphs or tables.

## 3. Results and Discussion

### 3.1. An Analysis of Thermal Properties of Biocomposite

**Figure 5:**
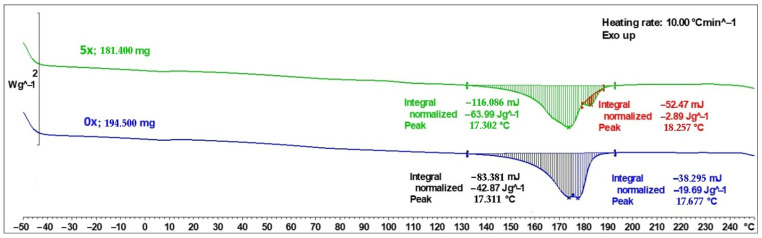
Heat flow versus temperature (DSC curves) for PHBV–SCG (45 wt%) composites: 0×—composite sample produced from virgin material (PHBV–SCG 45 wt%), 5×—composite sample reprocessed/recycled five times.

DSC curves for PHBV biocomposites containing 45 wt% SCG in their initial state (0×) and after five recycling cycles (5×) are presented in Figure 5. In both cases, a characteristic melting peak associated with the crystalline phase of the PHBV matrix was recorded; however, clear quantitative and qualitative differences in the thermal signals are evident.

For the initial sample (0×), the main melting peak was recorded at 173.11 °C, with a total melting enthalpy of −833.81 mJ, corresponding to a normalized value of 42.87 J·g^−1^. Additionally, a secondary, weaker signal was observed at a higher temperature (176.77 °C) with an enthalpy of −382.95 mJ (−19.69 J·g^−1^). The presence of this secondary peak may indicate heterogeneity of crystallites in the PHBV structure and the potential formation of multiple crystalline forms in the presence of a lignocellulosic filler [31,32,33].

For the biocomposite subjected to five recycling cycles (5×), the main melting peak shifted slightly to 173.02 °C, and its intensity increased substantially—the total melting enthalpy reached −1160.86 mJ, equivalent to −63.99 J·g^−1^ when normalized to sample mass. This reflects a noticeable increase in the crystalline phase content after repeated processing cycles. This phenomenon can be attributed to polymer chain degradation during repeated injection molding cycles—shortened PHBV macromolecules exhibit greater mobility and easier chain alignment, which promotes crystallization [34,35]. Additionally, an extra endothermic signal was recorded at 182.57 °C (−52.47 mJ; −2.89 J·g^−1^), which may be associated with recrystallization or the formation of a modified PHBV crystalline structure induced by the natural filler [36].

Analysis of the DSC thermograms therefore indicates significant changes in the material’s thermal structure. The primary melting peak remained near 173 °C, but an additional peak appeared at a higher temperature (~182.6 °C), suggesting the development of a more ordered or alternative crystalline form induced by the filler. This behavior is consistent with secondary crystallization mechanisms commonly reported for PHBV during heating, attributed to the coexistence of crystallites of varying perfection and to the melting–recrystallization–remelting process [31,32,33,37]. The total melting enthalpy recorded during the DSC analysis (Figure 5) for the PHBV 0× and 5x biocomposite samples is −62.56 and −66.89 J·g^−1^, respectively. These data refer to the mass of the entire sample subjected to analysis, while the melting peak is the result of melting of the PHBV polymer only. After taking into account the addition of 45% filler, the given values were recalculated not for 1 g of the biocomposite sample, but for 0.55 g of polymer. The corrected melting enthalpy values for the 0x sample are: −113.74 J·g ^−1^, and for the 5x sample: −121.66 J·g^−1^. These data were then used to calculate the degree of crystallinity *X*(%) using the procedure described in the paper [38], based on the relationship:

(1) $$X\left(\%\right)=\frac{∆H}{{∆H}_{100\%}}\cdot 100\%$$

where Δ*H* is the experimentally determined melting enthalpy, Δ*H*_100%_ is the melting enthalpy of the 100% crystallized polymer (for PHBV the value of 146 was assumed according to [38]). The calculated degrees of crystallinity for the melting enthalpies converted to PHBV are as follows: for 0x 77.9%, and for sample 5x 83.3%. This demonstrates an over 5% increase in the crystalline phase share in the 5x biocomposite, i.e., after subjecting sample 0x to five recycling cycles.

In the literature, it has been repeatedly emphasized that mechanical recycling of PHBV leads to macromolecular degradation, primarily through chain scission, which increases segment mobility and facilitates crystallization processes [34,35,39]. Dedieu et al. [39] demonstrated that successive processing cycles cause a systematic decrease in molecular weight, accompanied by increased crystallinity and a loss of mechanical properties, despite the relative stability of the melting temperature. Similar effects are also observed in other biopolymers, such as PLA and PBS, where increased crystallinity results in higher stiffness and hardness but simultaneously reduces tensile strength, elongation at break, and impact strength [40,41,42].

The obtained DSC results are consistent with the trends observed in the mechanical property tests described later in this work: increased crystallinity leads to a higher modulus of elasticity, while reducing resistance to plastic deformation and energy absorption. The increased brittleness of the material after multiple recycling cycles is therefore a natural consequence of intensified secondary crystallization and the reduced flexibility of the amorphous phase. Notably, the need to apply higher temperatures during successive injection molding cycles intensifies thermal degradation [43].

### 3.2. Evaluation of Mechanical Properties in the Uniaxial Tensile Test

The results of the uniaxial tensile test for the PHBV biocomposite containing 45 wt% SCG are presented in Table 6. Based on the obtained data, clear differences in the mechanical behavior of the samples were observed, depending on the number of reprocessing cycles.

Tensile Modulus (E_t_) showed an increasing trend with the number of recycling cycles. For the virgin material (0×), it averaged 3092.54 MPa, while after five reprocessing cycles it reached 3388.30 MPa, representing an increase of approximately 9.6%. The lowest modulus was recorded after the first cycle (3032.65 MPa); however, subsequent series showed a gradual increase in sample stiffness. This effect can be attributed to the shortening of PHBV macromolecular chains due to thermo-mechanical degradation and the intensification of secondary crystallization processes, which contribute to material stiffening.

The tensile strength (σ_m_) values remained relatively stable. The initial value of 16.54 MPa (0×) decreased slightly to 16.24 MPa (5×), corresponding to a reduction of around 1.8%. The lowest strength was observed after the second cycle (15.85 MPa), followed by a noticeable recovery in the 3x and 4x series. This fluctuation is likely attributed to the competition between opposing mechanisms occurring during reprocessing: polymer matrix degradation versus microstructural homogenization and secondary crystallization. The initial drop at 2x is likely driven by the onset of thermal degradation and PHBV chain scission. However, the subsequent increase in tensile strength suggests that the additional shear forces applied during the 3rd and 4th cycles facilitated the breakdown of remaining SCG agglomerates and improved filler distribution within the matrix. Simultaneously, the shortened polymer chains exhibited higher mobility, leading to an increase in the crystalline phase fraction (as confirmed by DSC analysis), which acts as a reinforcing domain. By the 5th cycle, the cumulative degradation of the polymer chains likely became the dominant factor, stabilizing the mechanical performance.

A different trend was observed for strain at tensile strength (ε_m_), which showed a clear downward tendency. For the virgin material, the average strain was 1.28%, whereas after five recycling cycles it decreased to 1.05%, representing a reduction of approximately 18%. The most significant drop occurred after the fourth cycle, when strain fell to 1.06%. This effect can be attributed to the increased crystallinity of the PHBV matrix and the degradation of polymer chains, leading to a reduced proportion of the amorphous phase responsible for plastic deformation.

The obtained results clearly indicate that repeated processing of PHBV–SCG biocomposites increases the material’s stiffness while reducing its deformability. The relatively constant tensile strength at maximum stress suggests that matrix degradation does not significantly reduce tensile strength but instead leads to increased brittleness. These findings are consistent with previous DSC analyses and the observed decreases in impact tensile strength and hardness, highlighting the progressive structural degradation of the polymer and the increasing crystalline phase content in successive recycling cycles.

The tensile test results obtained in this study for the PHBV–SCG (45 wt%) biocomposite after multiple processing cycles are consistent with trends reported for similar polymer–biofiller systems. Research by Janowski et al. [10] on PHBV–buckwheat hull composites demonstrated stabilization of mechanical parameters as a result of improved structural homogeneity in successive processing cycles. Despite an initial decrease in some properties, an increase in stiffness was observed, attributed to secondary crystallization—analogous to the increase in Young’s modulus reported in this work. Similarly, Zhao et al. [7] studied PHBV-based composites with untreated SCG and found that while elongation at break decreased (down to 43%), tensile modulus and strength remained relatively stable compared to neat PHBV. This indicates that polymer matrix degradation does not necessarily lead to a significant loss in tensile strength but primarily affects plastic properties—a finding also confirmed in our study, where maximum stress remained stable while ductility decreased. Boughanmi et al. [44] investigated the effect of repeated processing on PLA–SCG (5 wt%) composites, reporting a significant reduction in both tensile strength and elongation at break, attributed to chain scission and weak interfacial adhesion. However, they did not observe a stiffness improvement, likely due to the lower filler content and different PLA matrix properties compared to PHBV. In contrast, Suaduang et al. [45] examined PLA composites containing 5–30% SCG and reported that lower filler levels (≤10%) enhanced stiffness without significantly compromising strength, whereas higher loadings introduced agglomerates and defects that reduced ductility—a trend similar to our observations at 45% SCG in PHBV. Rocha et al. [46] analyzed PHB composites with SCG and found that moderate filler contents (15%) preserved good strength and stiffness, whereas higher levels caused plasticity loss and increased brittleness, which aligns with the findings of this study. Reis et al. [47] reported that PHB composites with 20% coffee parchment exhibited substantial increases in tensile modulus and strength, attributed to the filler’s reinforcing effect. However, as in this study, a reduction in ductility was observed, indicating greater brittleness. Finally, Morreale et al. [48] investigated the influence of mechanical recycling on biopolymer–wood flour green composites, noting an increase in stiffness and a maintenance of low elongation at break after reprocessing. They attributed this stiffening effect to improved filler dispersion and polymer chain scission, a behavior that correlates with the structural changes we observe in the PHBV matrix.

The evaluation of the mechanical behavior of the coffee-ground-filled composites shows that both the presence of the filler and its concentration strongly influence the strength characteristics of the material. At this point, it is reasonable to compare the obtained results with literature data for comparable systems based on a PHBV matrix or neat PHBV [7,36,37]. Unfilled PHBV is characterized by an elastic modulus of 2617 MPa and a tensile strength of 35 MPa, with a maximum elongation at break of approximately 4%. The incorporation of coffee grounds leads to pronounced modifications—already at a filler loading of 15 wt%, the elastic modulus rises to 3308 MPa, which indicates a substantial increase in material stiffness. This enhancement is likely attributed to the presence of irregular, highly jagged coffee particles dispersed in the matrix, which restricts its deformability and thus stiffens the composite. Owing to their complex morphology, coffee grounds appear to behave as a micro-reinforcing phase, generating local obstacles to matrix deformation and thereby increasing the rigidity of the composite. However, this reinforcing capability diminishes as the filler content continues to grow—at 30 wt% and 45 wt% coffee grounds, the elastic modulus reaches 3085 MPa and 3092 MPa, respectively, suggesting that the stiffening effect of the coffee filler in the PHBV matrix may have reached a saturation point.

### 3.3. Hardness Assessment

**Figure 6:**
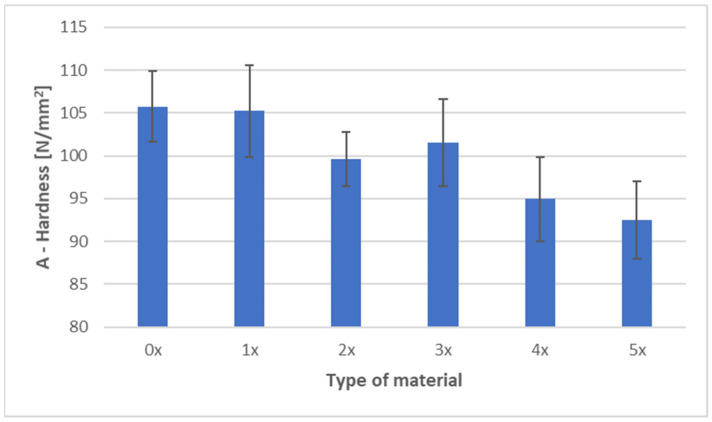
Hardness of PHBV–SCG (45 wt%) biocomposites after multiple processing cycles (area A).

**Figure 7:**
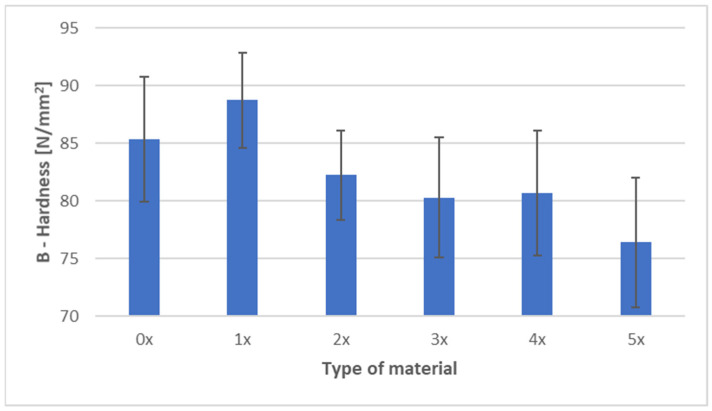
Hardness of PHBV–SCG (45 wt%) biocomposites after multiple processing cycles (area B).

Figure 6 and Figure 7 present the ball indentation hardness test results for PHBV–SCG (45 wt%) biocomposites, both in the virgin state (0×) and after five reprocessing cycles (1×–5×). Measurements were conducted in two characteristic regions of the dumbbell-shaped tensile specimens: the central gauge section (area A) and the grip section (area B).

In area A (gauge section of the dumbbell specimen), a clear downward trend in hardness values was observed with an increasing number of processing cycles. The initial value of 105.75 N/mm^2^ (0×) systematically decreased, reaching 92.50 N/mm^2^ after five recycling cycles (5×), representing a reduction of approximately 12.5%. The lowest values were recorded for samples after four and five reprocessing cycles, with differences exceeding three times the standard deviation, which makes these changes significant from a material analysis perspective. A slight increase in hardness after the third cycle (101.54 N/mm^2^) may be attributed to partial structural ordering and secondary crystallization, although this effect did not compensate for the cumulative degradation observed in subsequent cycles.

In area B (grip section of the dumbbell specimen), the trend was more complex. After the first recycling cycle, hardness increased to 88.73 N/mm^2^ (an increase of approximately 4% compared to the 0× sample), likely due to localized filler particle refinement and improved dispersion within the matrix. However, subsequent cycles caused a gradual decrease in hardness, reaching 76.40 N/mm^2^ after five cycles, corresponding to a 10.5% reduction compared to the virgin material. Variability in area B (coefficient of variation at 6–7%) was notably higher than in area A (4–5%), reflecting greater heterogeneity in the grip section caused by differences in flow and cooling conditions during injection molding.

Comparing both regions, hardness values in the gauge section (A) were consistently higher than in the grip section (B), regardless of the number of recycling cycles. The difference ranged from 14 to 21 N/mm^2^ and fluctuated slightly depending on the degree of reprocessing, indicating localized variations in material structure and a possible influence of orientation-induced stresses. The results confirm that repeated processing leads to the deterioration of the mechanical properties of the biocomposite, as evidenced by a consistent decline in hardness in both analyzed areas. This finding aligns with DSC results, which showed an increased crystalline phase fraction due to polymer chain degradation and enhanced secondary crystallization. While this phenomenon contributes to material stiffness, it simultaneously reduces its resistance to localized deformation and increases brittleness, as demonstrated by the analyzed hardness results.

The obtained results are consistent with literature reports on the impact of mechanical recycling on the mechanical properties of biocomposites containing SCG. Studies on PLA composites with 5 wt% SCG demonstrated that successive processing cycles lead to a deterioration of mechanical properties, such as tensile strength and elongation at break. At the same time, an increase in hardness was observed, attributed to enhanced crystallinity resulting from polymer chain degradation and the presence of the filler [44]. Although the dominant effect in this study was an overall decrease in hardness, the slight increase observed after the third cycle (area A) may be related to a similar secondary crystallization phenomenon; however, ongoing degradation prevailed in subsequent cycles. Similarly, Janowski et al. [1] compared PHBV composites containing various bio-fillers, including SCG, and found that although filler incorporation increases material stiffness, it does not improve mechanical strength due to weak interfacial adhesion. These findings align with the present study, where the filler’s high loading (45 wt%) did not compensate for matrix degradation. In another study on PHBV–buckwheat hull composites subjected to five reprocessing cycles, an initial decline in mechanical properties was followed by stabilization. This was attributed to improved microstructural homogeneity and better filler dispersion after multiple processing cycles [10]. In contrast, the current research shows a more linear decrease in hardness, which may indicate a stronger effect of chemical degradation at high filler content or less effective filler dispersion. Furthermore, Majrashi et al. [6] reported that although SCG can be successfully dispersed in a PHBV matrix, particle aggregation at higher concentrations leads to void formation and weaker interfacial adhesion, negatively affecting mechanical properties and increasing result variability. In light of these observations, the higher variability of hardness in area B could be attributed to localized heterogeneities in particle distribution and differences in molding conditions.

### 3.4. Impact Tensile Strength Assessment

**Figure 8:**
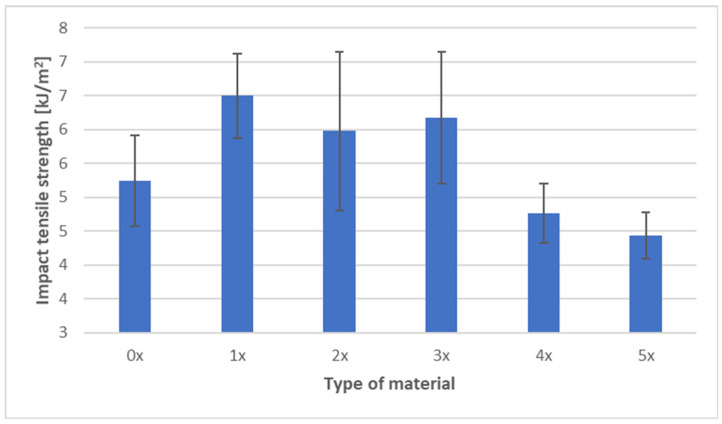
Impact tensile strength of PHBV–SCG (45 wt%) biocomposites after multiple processing cycles.

The results of tensile impact strength testing for the PHBV biocomposite with 45 wt% spent coffee grounds (SCG) are presented in Figure 8.

For the virgin material (0×), the average tensile impact strength was 5.24 kJ·m^−2^. After the first recycling cycle, a significant increase was observed, reaching 6.50 kJ·m^−2^, which represents an improvement of approximately 24% compared to the reference sample. Relatively high values were also maintained after the second (5.98 kJ·m^−2^) and third (6.18 kJ·m^−2^) processing cycles. The maximum tensile impact strength was recorded after the first cycle, with stabilization at an elevated level observed through the third cycle of reprocessing.

In subsequent processing stages, however, a decline in impact resistance was observed. After the fourth cycle, the value decreased to 4.76 kJ·m^−2^, and after the fifth cycle it dropped further to 4.44 kJ·m^−2^, representing a reduction of about 15% relative to the virgin state. These results indicate that mechanical recycling initially enhances the impact resistance of the biocomposite, but this effect is short-lived and is eventually negated by progressive degradation of the polymer matrix in later cycles.

The observed trends can be explained by microstructural processes. In the early cycles, the improvement in tensile impact strength likely resulted from secondary crystallization of the PHBV matrix and better dispersion of filler particles achieved through repeated melting and mixing, which improved crack energy dissipation. In the later cycles, polymer chain scission and weakening of interfacial adhesion reduced the material’s ability to absorb energy, increasing its brittleness.

These findings align with the general trend observed in biocomposites subjected to multiple reprocessing cycles: modest improvements in mechanical performance may appear in early cycles due to microstructural rearrangement, but chemical degradation of the matrix and the accumulation of microstructural defects ultimately dominate, leading to a reduction in impact resistance over time.

The observed increase in tensile impact strength during the initial recycling cycles (1×–3×), followed by a decline after subsequent reprocessing, aligns with trends reported in the literature for PHBV-based and other biodegradable polymer biocomposites. In their study on PHBV composites with buckwheat hulls, Janowski et al. [10] demonstrated that after an initial reduction in some mechanical properties, further processing cycles led to parameter stabilization due to improved microstructural homogeneity and better filler embedding in the matrix. This could explain the stabilization of tensile impact strength at an elevated level observed in this study up to the third cycle, before material degradation became dominant. Similarly, Majrashi et al. [6] showed that chemical surface modification of SCG particles improves their adhesion to the PHBV matrix, resulting in enhanced mechanical resistance. In this work, the absence of such modification may have contributed to the decrease in impact strength during later recycling cycles, as interfacial bonding progressively weakened. Studies on PLA composites with 5% SCG also reported that repeated recycling cycles caused deterioration of strength properties, including tensile strength and impact resistance, despite increased stiffness [44]. The authors attributed this to polymer chain degradation and matrix quality deterioration, directly affecting energy absorption capacity—consistent with our results after the 4× and 5× cycles. Moreover, Rocha et al. [46] reported that PHB composites with deoiled SCG retained good ductility and impact resistance at moderate filler levels (up to 15%), whereas higher loadings led to agglomerates and defects, reducing energy dissipation capacity. Based on these findings, the high SCG content (45%) in our biocomposite likely exacerbates the accumulation of structural defects during prolonged processing. Additionally, Boughanmi et al. [44] observed that while multiple processing cycles of PLA–SCG composites generally decreased mechanical performance, initial stages sometimes showed improved impact resistance due to secondary crystallization and system homogenization before degradation effects prevailed. Zhao et al. [7] confirmed that PHBV composites with 10–30% SCG exhibited limited ductility and increased brittleness in the absence of filler surface modification. The observed decline in tensile impact strength after the fourth and fifth cycles in our study may result from similar phenomena—reduced interfacial adhesion and defect accumulation. Furthermore, de Bomfim et al. [49] found that PLA–SCG impact strength decreased with rising SCG content, attributing this to reduced material capacity for local deformation and energy absorption, especially at higher loadings. A comparable effect is evident in this study, where SCG content reaches 45%.

### 3.5. The Shrinkage Determination

**Figure 9:**
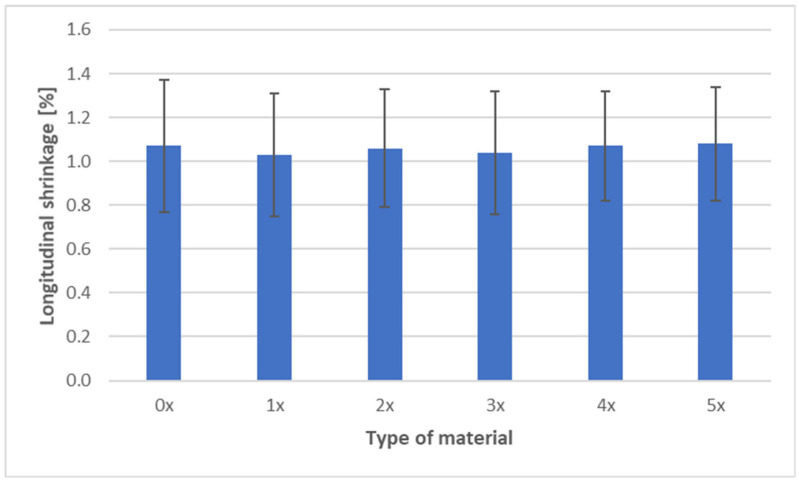
Longitudinal shrinkage of PHBV–SCG (45 wt%) biocomposites after multiple processing cycles.

**Figure 10:**
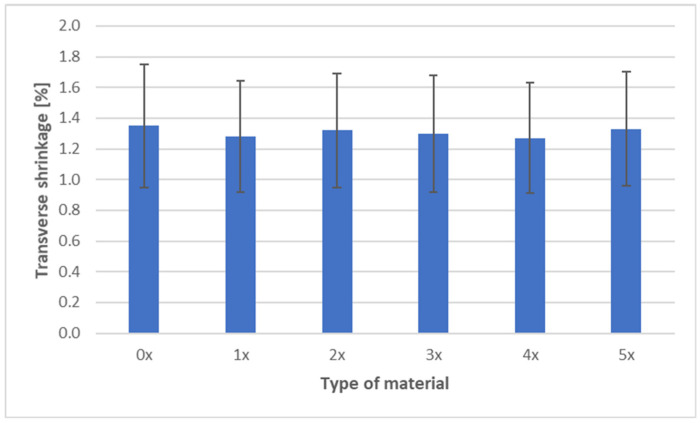
Transverse shrinkage of PHBV–SCG (45 wt%) biocomposites after multiple processing cycles.

**Figure 11:**
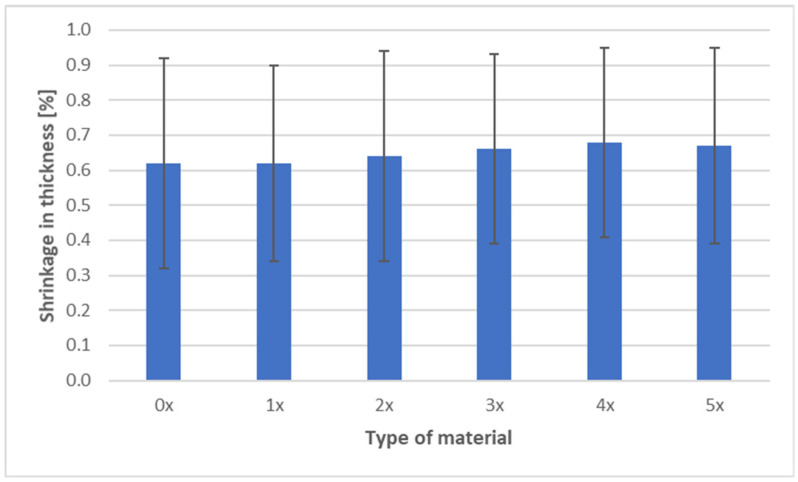
Shrinkage in thickness of PHBV–SCG (45 wt%) biocomposites after multiple processing cycles.

The shrinkage measurements of PHBV biocomposites containing 45 wt% SCG after successive recycling cycles are presented in three characteristic directions: longitudinal, transverse, and through-thickness (Figure 9, Figure 10 and Figure 11).

The longitudinal shrinkage remained nearly constant, within the range of 1.03–1.08%, throughout all recycling cycles. The absence of significant changes along this direction suggests structural stability of the composite in the flow direction. This behavior can be attributed to the geometry of the SCG particles—their irregular, isotropic shape (10–300 µm, with a porous and rough surface) does not promote longitudinal orientation during material flow. Unlike hemp or flax fibers, which tend to align along the flow stream and strongly influence shrinkage in this direction, SCG particles act more as an isotropic filler, stabilizing but not significantly altering shrinkage [1,7].

The transverse shrinkage showed a clear decreasing trend, from 1.35% (0×) to 1.27% (4×), followed by a slight increase to 1.33% (5×). This reduction can be explained by improved “anchoring” of SCG particles in the matrix after successive recycling cycles, as particle fragmentation and more uniform redispersion occur. The porous structure and rough edges of SCG particles increase interfacial friction, which limits later stress relaxation. This effect is supported by other studies, which reported enhanced interfacial bonding due to biological treatments or modified filler topology [6,15,16].

The most notable phenomenon is the increase in thickness shrinkage, which rose from 0.62% (0×) to 0.68% (4×), followed by stabilization at 0.67% (5×). This effect results from changes in PHBV crystallization caused by chain degradation—shorter macromolecules are able to organize more rapidly during cooling, leading to greater contraction along the thickness direction. The irregular SCG particles do not create barriers that could limit this shrinkage—unlike wood or fiber reinforcements, which can act as anisotropic barriers [48,50]. Similar behavior was also observed by other researchers, who reported intensified shrinkage phenomena and reduced mechanical properties at higher SCG contents [51].

Analysis of shrinkage results in the context of mechanical properties indicates a consistent relationship. The stability of longitudinal shrinkage corresponds to relatively stable values of elastic modulus, SCG particles, while increasing stiffness (due to their deformation-blocking effect), does not change anisotropy along the flow direction [7]. The reduction in transverse shrinkage is reflected in hardness measurements, as the composite exhibits a more uniform structure and improved load transfer [6,18,19]. In contrast, the increase in thickness shrinkage correlates with reduced impact strength and tensile strength—greater contraction promotes the formation of defects and micropores, as confirmed by morphological observations, explaining the decreased resistance to fracture [40]. In summary, the effect of SCG on the shrinkage of PHBV biocomposites should be interpreted in the context of their morphology. The irregular, porous particles act as micro-reinforcement in the plane of the molded part, stabilizing transverse shrinkage, but they do not counteract the increase in thickness shrinkage. This behavior may be critical when designing components requiring high-dimensional stability and mechanical performance [52].

### 3.6. Microstructure Evaluation at Fractures

**Figure 12:**
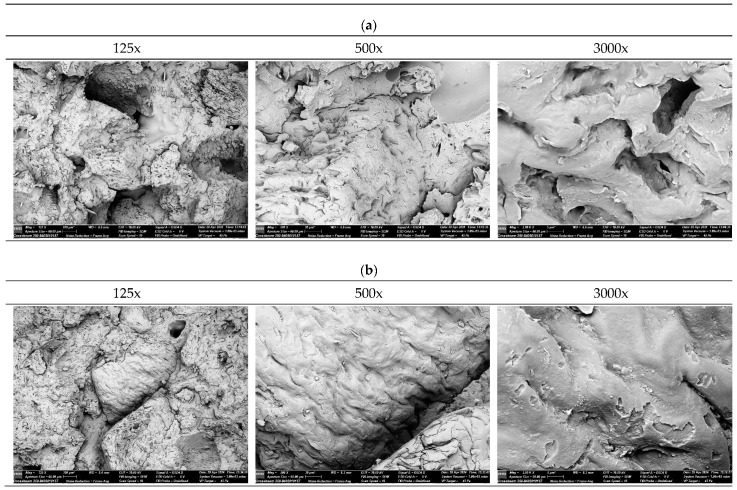
SEM images of PHBV-SCG (45 wt%) biocomposites after multiple processing cycles: (**a**) 0x, (**b**) 5x).

Figure 12 presents fracture surface images of PHBV–SCG (45 wt%) biocomposites for samples in their original state (0×) and after five recycling cycles (5×), obtained using SEM at magnifications of 125×, 500×, and 3000×. Detailed analysis enables the assessment of interfacial adhesion quality, filler dispersion level, and fracture mechanisms.

At low magnification (125×), the fracture surface appears irregular and porous, with numerous pores and voids within the matrix. In many areas, agglomerates of irregularly shaped SCG particles are observed, indicating limited dispersion efficiency during processing. At 500× magnification, interfacial regions between the SCG filler and the PHBV matrix become visible, showing gaps and microvoids around the particles. This phenomenon indicates weak interfacial adhesion and local debonding of the matrix from the filler. The highest magnification (3000×) reveals detailed failure mechanisms: numerous empty impressions left by pulled-out particles, as well as microcracks propagating along phase boundaries. The observed fracture surfaces indicate a mixed failure mode—partly ductile (with signs of matrix deformation around larger particles) and partly brittle (with abrupt, smooth fracture facets).

The fracture surfaces of samples after five recycling cycles reveal significant morphological changes. At 125× magnification, the dispersion of SCG particles within the matrix appears more uniform, and the number of large voids is reduced. This effect can be attributed to repeated melting and shearing, which promote agglomerate breakdown and a more homogeneous filler distribution. At 500× magnification, the structure shows lower porosity than the virgin material, but numerous straight microcracks are present, indicating a dominant brittle fracture mechanism. In many regions, the fracture surface appears smoother, suggesting reduced ability of the matrix to undergo local deformation. At 3000× magnification, numerous smooth fracture facets and microcracks propagating along both the interfacial boundaries and through the matrix are visible. The lack of evident ductile deformation around the filler particles, combined with the predominance of flat, smooth surfaces, confirms the brittle nature of failure. This indicates that thermomechanical degradation of the PHBV matrix and its increased crystallinity limit the material’s energy absorption capability and promote crack propagation along structural defects.

The comparison of SEM images of 0x and 5x samples indicates noticeable microstructural evolution. While quantifying the exact degree of dispersion based solely on fracture surfaces presents challenges, the 5x samples exhibit a reduction in the size and frequency of large voids and extensive particle pull-outs compared to the pristine material (0x). This morphological change suggests that the repeated shear forces acting on the melt during recycling cycles may facilitate the breakdown of larger SCG agglomerates, leading to potentially improved homogenization. However, the most prominent feature in the 5x samples is the transition towards a brittle fracture mechanism. The prevalence of smooth fracture facets and matrix microcracking confirms that the enhanced crystallinity and thermal degradation of the PHBV matrix play a dominant role in the material’s failure behavior after multiple reprocessing steps. The micromorphological changes observed in this study are consistent with findings reported in the literature for PHBV-based and other biodegradable polymer composites. Zhao et al. [7] demonstrated that PHBV composites containing untreated SCG exhibit poor interfacial adhesion, visible as voids around particles and irregular agglomerates, leading to easy particle pull-out. These findings are confirmed by the 0× sample analysis, which showed clear pull-out effects and structural voids negatively affecting ductility and impact resistance. Rocha et al. [46], in studies of PHB composites with defatted SCG, observed that high filler content (>15%) leads to disrupted phase continuity and morphological defects, particularly after prolonged processing. This aligns with the present analysis of 5× recycled samples, where improved dispersion was accompanied by increased microcrack density and a dominant brittle fracture mechanism. Similarly, Boughanmi et al. [44] reported that PLA–SCG composites developed microcracks and weakened interfacial bonding after just a few recycling cycles. Their work showed that repeated melting led to matrix degradation and a loss of energy absorption capability—findings consistent with the present results. Moreover, Reis et al. [47] noted that PHB composites containing coffee waste particles with a high surface area exhibited improved stiffness but were prone to crack propagation along the filler–matrix interface. The smooth fracture surfaces and microcracks seen in recycled (5×) samples in this study provide strong confirmation of this effect. Overall, the SEM-documented microstructural changes—such as improved filler dispersion, reduced void content, and increased brittleness—correlate closely with earlier studies and provide direct evidence of the degradation and crystallization phenomena that occur during repeated processing of PHBV–SCG biocomposites.

## 4. Conclusions

This study provides a comprehensive evaluation of the effects of multiple mechanical recycling cycles (up to five) on the structure, processing behavior, and properties of PHBV–SCG (45 wt%) biocomposites.

DSC analysis revealed a systematic increase in crystallinity (from ~43 to ~64 J·g^−1^) after successive processing cycles and the appearance of an additional melting peak (~182 °C), confirming secondary crystallization and PHBV chain reorganization.

Tensile testing demonstrated an increase in stiffness (Young’s modulus +9.6% after five cycles), with tensile strength remaining relatively stable (−1.8%) and elongation at break decreasing by ~18%, indicating increased brittleness caused by chain degradation and higher crystallinity. Hardness measurements showed a consistent decrease in both analyzed specimen areas, confirming reduced local deformation resistance and growing brittleness. Impact tensile strength initially increased (+24% after the first cycle), attributed to improved filler dispersion, but subsequently declined, reflecting the transition from microstructural homogenization to matrix degradation.

Shrinkage analysis revealed stable longitudinal shrinkage, reduced transverse shrinkage, and a slight increase in thickness shrinkage, illustrating the anisotropic influence of SCG particles.

SEM observations confirmed improved filler distribution and reduced porosity after multiple processing cycles but highlighted a dominant brittle fracture mechanism and interfacial debonding, correlating with decreased impact resistance.

Overall, the findings demonstrate that PHBV–SCG composites, even with a high filler content (45 wt%), can withstand multiple injection molding cycles while maintaining acceptable mechanical performance and dimensional stability, making them promising candidates for circular economy applications.

As an extension of this work, future research will focus on evaluating the long-term performance of PHBV–SCG biocomposites under cyclic and time-dependent loading, including fatigue and creep tests, to better understand their durability in real application scenarios. Additional studies will address surface modification of SCG to enhance interfacial adhesion, as well as advanced structural characterization (DMA, FTIR, NMR) and environmental degradation assessments. These efforts will allow for a more complete identification of the material’s limitations and optimization pathways, further supporting the development of high-filler PHBV–SCG biocomposites for circular and sustainable material applications.

## Figures and Tables

**Table 1:** Designations of the manufactured and tested samples.

Series Designation	Description
**0x**	Sample made from virgin material obtained by injection molding
**1x**	Sample obtained by recycling (grinding and reprocessing) the 0× sample
**2x**	Sample obtained by recycling (grinding and reprocessing) the 1× sample
**…**	…
**5x**	Sample obtained by recycling (grinding and reprocessing) the 4× sample

**Table 2:** Set temperatures for individual heating zones of the single-screw extruder.

Head [°C]	Zone 3 [°C]	Zone 2 [°C]	Zone 1 [°C]	Feed Hopper Zone [°C]	Screw Speed [rpm]
157	155	150	148	33	100

**Table 3:** Processing parameters for injection molding of the primary biocomposite (0×).

Cooling Time [s]	20
Packing time [s]	25
Packing pressure [MPa]	30
Mold temperature [°C]	80
Injection speed [cm^3^/s]	35
Melt temperature	170
Injection pressure	110

**Table 4:** Processing parameters for injection molding of specimens during successive reprocessing cycles (1×–5×).

Type of Material	1x	2x	3x	4x	5x
Cooling time [s]	20	20	20	20	20
Packing time [s]	25	25	25	25	25
Packing pressure [MPa]	30	30	30	30	30
Mold temperature [°C]	80	80	80	80	80
Injection speed [cm^3^/s]	35	40	40	45	45
Melt temperature	173	173	176	176	180
Injection pressure	110	110	110	110	110

**Table 5:** Experimental design and test matrix.

Operation/Series	0x	1x	2x	3x	4x	5x
Processing: Extrusion	✓	-	-	-	-	-
Processing: Injection Molding	✓	✓	✓	✓	✓	✓
Linear Shrinkage	✓	✓	✓	✓	✓	✓
Static Tensile Test	✓	✓	✓	✓	✓	✓
Hardness Test	✓	✓	✓	✓	✓	✓
Tensile Impact Test	✓	✓	✓	✓	✓	✓
DSC Analysis	✓	-	-	-	-	✓
SEM Analysis	✓	-	-	-	-	✓

Note: ✓ denotes performed test; - denotes test not performed for this specific cycle.

**Table 6:** Results of static tensile tests for PHBV–SCG composites after multiple processing cycles.

Type of Filler	E_t_ [MPa]	σ_m_ [MPa]	ε_m_ [%]
0x (x *)	3092.54	16.54	1.28
s **	36.79	0.21	0.02
1x (x)	3032.65	15.89	1.19
s	53.80	0.25	0.04
2x (x)	3101.49	15.85	1.17
s	88.68	0.09	0.04
3x (x)	3125.80	16.04	1.18
s	77.99	0.12	0.08
4x (x)	3313.98	16.20	1.05
s	91.91	0.16	0.04
5x (x)	3388.30	16.24	1.05
s	72.65	0.11	0.02

* x—the average value. ** s—standard deviation.

## Data Availability

The original contributions presented in the study are included in the article, further inquiries can be directed to the corresponding authors.
